# Tumor diagnosis recharacterization enabled by comprehensive genomic profiling to guide precision medicine strategy

**DOI:** 10.1038/s41698-025-00942-5

**Published:** 2025-05-21

**Authors:** Ann Carr, Jennifer B. Jackson, Chris Coldren, Pranil Chandra, Faezeh Koohestani, Michelle Shiller, Robert Auber

**Affiliations:** 1 PathGroup, Nashville, TN 37217 USA; 2 Labcorp, Baltimore, MD 21224 USA

**Keywords:** Cancer genomics, Cancer of unknown primary, Tumour biomarkers, Diagnostic markers, Predictive markers, Prognostic markers, Cancer

## Abstract

Comprehensive genomic profiling (CGP) via next-generation sequencing is standard clinical practice for advanced and metastatic cancers in the U.S. and can help identify clinically actionable alterations in patients who may benefit from targeted therapies. CGP can also complement clinicopathological findings and in certain cases, may lead to diagnostic recharacterization resulting in more precise therapeutic strategies. Here, we highlight examples where molecular findings resulted in tumor re-evaluation and subsequent recharacterization. Twenty-eight cases where CGP results were inconsistent with initial pathological diagnosis and clinical presentation were selected for secondary clinicopathological review to explore alternative diagnostic explanations more consistent with the genomic results. Genomic profiling identified clinically actionable and prognostic variants leading to more accurate therapeutic recommendations based on the updated diagnoses highlighting the value of CGP beyond biomarker detection for therapy selection and supporting its complementary use in diagnostic confirmation to unveil opportunities for precision medicine strategies.

## Introduction

The pathology work-up to determine a primary diagnosis for a cancer patient is generally initiated by tumor characterization through physical examination, radiographic imaging, tissue biopsy, and laboratory testing, followed by confirmatory results from molecular analysis^1^. Testing for single-gene alterations (DNA) or expression markers (RNA), or protein-specific biomarkers often includes immunohistochemistry (IHC), fluorescence in situ hybridization (FISH), and/or polymerase chain reaction (PCR), depending on clinical guideline recommendations and the established standard of care for each indication^2^. Despite the availability and clinical value of these testing modalities, the need for cost-effective and simultaneous multi-gene interrogation has driven the adoption of more complex genomic methodologies, particularly next-generation sequencing (NGS)^3–6^. Comprehensive molecular profiling, defined within this study as an NGS test consisting of a large panel of genes (>500) that is able to simultaneously detect a wide variety of somatic genomic alterations and molecular signatures from DNA^7^, optimizes the use of tissue specimen, offers breadth of coverage, and aligns with current guidelines set by the National Comprehensive Cancer Network (NCCN), American Society of Clinical Oncology (ASCO), and Association for Molecular Pathology (AMP) for most indications^8^, with support from individual professional societies specific to tumor site in many instances (CAP/IASLC/AMP, WHO, SGO, etc.). NGS-based assays are increasingly employed by pathologists to complement their morphologic and molecular assessment of tumors in making the final diagnostic determination^9,10^, given our improved understanding of the cancer genome landscape across tumor types^11^. However, the seamless integration of NGS-based assays into the clinical care continuum requires undisrupted flow of tumor tissue from biopsy to triage for relevant analyses as well as aggregation and interpretation of complex results^12^. It is therefore imperative that pathologists are incorporated as leaders within the multidisciplinary team responsible for patient care and that they also stay abreast of the latest advancements in the field.

With the incorporation of NGS as a clinical tool enabling precision medicine, more patients are gaining access to comprehensive genomic profiling (CGP) to identify clinically relevant and actionable biomarkers and signatures^13–15^. The collected information is then used to predict prognosis, guide US Food and Drug Administration (FDA)-approved therapy selection based on biomarker findings, and identify opportunities for clinical trial enrollment^16^. In fact, the prospective NGS evaluation of >900 advanced solid tumor cases from a completed clinical trial suggested that high-throughput genomic analysis could improve clinical outcomes for a subset of patients with hard-to-treat cancers^17^. Similarly, a real-world observational study with patient follow-up demonstrated a positive correlation between NGS-based categorization of patients and improved clinical outcomes in initial management of advanced or metastatic disease in certain indications with multiple molecular markers as well as in rare cancers, and for clinical trial screening^18^.

Interestingly, the widespread adoption of NGS in the clinical settings has, in rare cases, revealed inconsistencies between the primary diagnosis and the diagnosis determined by NGS results triggering a secondary comprehensive review of all pathological findings to reconcile the discordance^19–21^. While relatively infrequent, these integrated reviews can result in (1) tumor reclassification, resulting in a change from one distinct indication to another, or (2) tumor refinement, where cancers of unknown primary (CUP) origin are assigned a more definitive tumor classification^22–25^. While these are rare events, in many instances, such recharacterization also offers therapeutic benefit allowing a patient to meet FDA approval criteria for certain biomarkers that also have a diagnostic role. In a published case report, NGS testing helped correct an inaccurate primary diagnosis of leiomyosarcoma to liposarcoma. Following tumor reclassification, the patient received an indication-matched treatment and exhibited clinical benefit, including improved progression-free survival (PFS) and quality of life^26^. Similarly, whole exome sequencing (WES) and RNA sequencing identified a molecular target for a CUP patient, leading to a tailored treatment with substantial radiological response across all tumor sites^27^. Finally, in a retrospective cohort of astroblastoma cases, comprehensive molecular profiling identified adult- and adolescent-specific, actionable molecular features leading to reclassification of the majority of cases into distinct diagnostic subgroups linked to approved therapies^20^. Overall, tumor reclassification provided patients with more accurate treatment regimens which improved treatment response and clinical outcomes and reduced unnecessary costs and the burden of repeated cycles of failed therapy.

The application of NGS to aid in tumor diagnostic refinement is best highlighted in CUP cases as it provides clear clinical utility by enabling greater access to targeted therapies^28,29^. Comprising 3%-5% of all malignancies, CUP is a heterogeneous group of metastatic tumors that are defined by the absence of a clinically known tissue of primary origin^29–34^. Patients with tumors classified as CUP are characterized by late clinical presentation and metastasis at or quickly after presentation with primary disease. Due to limited availability of treatment options for these ambiguous diagnoses, these patients have poor prognoses with a poor median overall survival (OS), ranging from 6 to 15 months, and limited access to empirical treatment options^22,35,36^. Refining CUP classification can therefore remove the ambiguity around the site of origin and may provide patients with greater access to precision oncology paradigms. In a study by Varghese et al, NGS analysis helped identify potentially targetable genetic alterations in 30% of analyzed cases of CUP^37^. Similarly, utilizing two complementary gene panels allowed for the identification of variants in CUP cases that were aligned with known oncogenic driver mutations and approved therapies^38^. In a recent case report, a CUP case was refined to metastatic breast cancer based on NGS results, offering access to a targeted therapy with a desirable outcome^39^. Lastly, generating molecular diagnostic classifiers based on NGS can inform treatment decisions, as demonstrated in 81.3% of 289 cases of CUP^24^.

The aim of this study is to examine and provide expanded evidence for the novel application of comprehensive genomic profiling (CGP) as a confirmatory diagnostic tool for tumor characterization to help improve therapeutic decisions and clinical outcomes. Using the Endeavor NGS test, which is powered by the Personal Genome Diagnostics (PGDx) elio™ tissue complete FDA-cleared assay^7^, we selected 28 cases with NGS findings not fully aligned with the initial primary diagnosis. Re-evaluation of the primary diagnoses using an integrated review strategy, resulting in the subsequent reclassification or refinement of these cases, led to more accurate therapeutic recommendations based on the updated diagnoses.

## Results

### CGP-driven disease reclassification and refinement

**Fig. 1:**
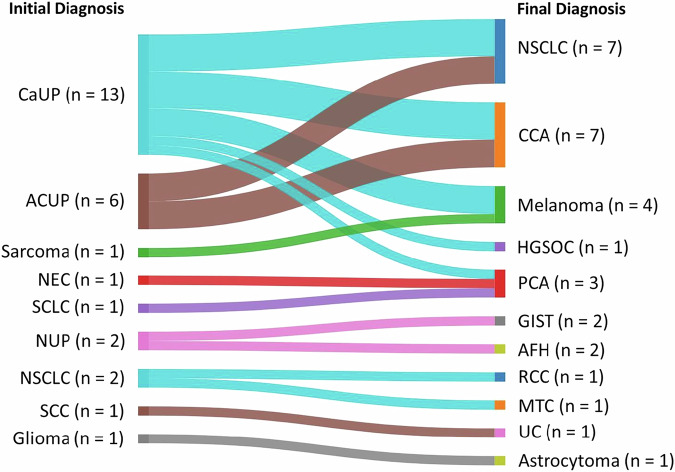
Summary of patient cases that underwent diagnostic recharacterization. Twenty-one CUPs, including CaUPs (*n* = 13), ACUPs (n = 6), and NUPs (*n* = 2), were refined to final diagnoses of NSCLC (*n* = 7), CCA (*n* = 7), melanoma (*n* = 3), HGSOC (*n* = 1), PCA (n = 1), GIST (*n* = 1), and AFH (*n* = 1). Seven cases with indication-specific initial diagnoses were reclassified to final secondary diagnoses of melanoma (*n* = 1), PCA (*n* = 2), RCC (*n* = 1), MTC (*n* = 1), UC (*n* = 1), and diffuse astrocytoma (*n* = 1). CAUP Carcinoma of unknown primary, ACUP Adenocarcinoma of unknown primary, NEC Neuroendocrine carcinoma, SCLC small cell lung cancer, NUP malignant neoplasm of unknown primary, NSCLC Non-small cell lung cancer, SCC Squamous cell carcinoma, CCA Cholangiocarcinoma, HGSOC High-grade serous ovarian cancer, PCA Prostate carcinoma, GIST Gastrointestinal stromal tumor, AFH Angiomatoid Fibrous Histiocytoma, RCC Renal cell carcinoma, MTC Medullary thyroid carcinoma, UC Urothelial carcinoma.

**Fig. 2:**
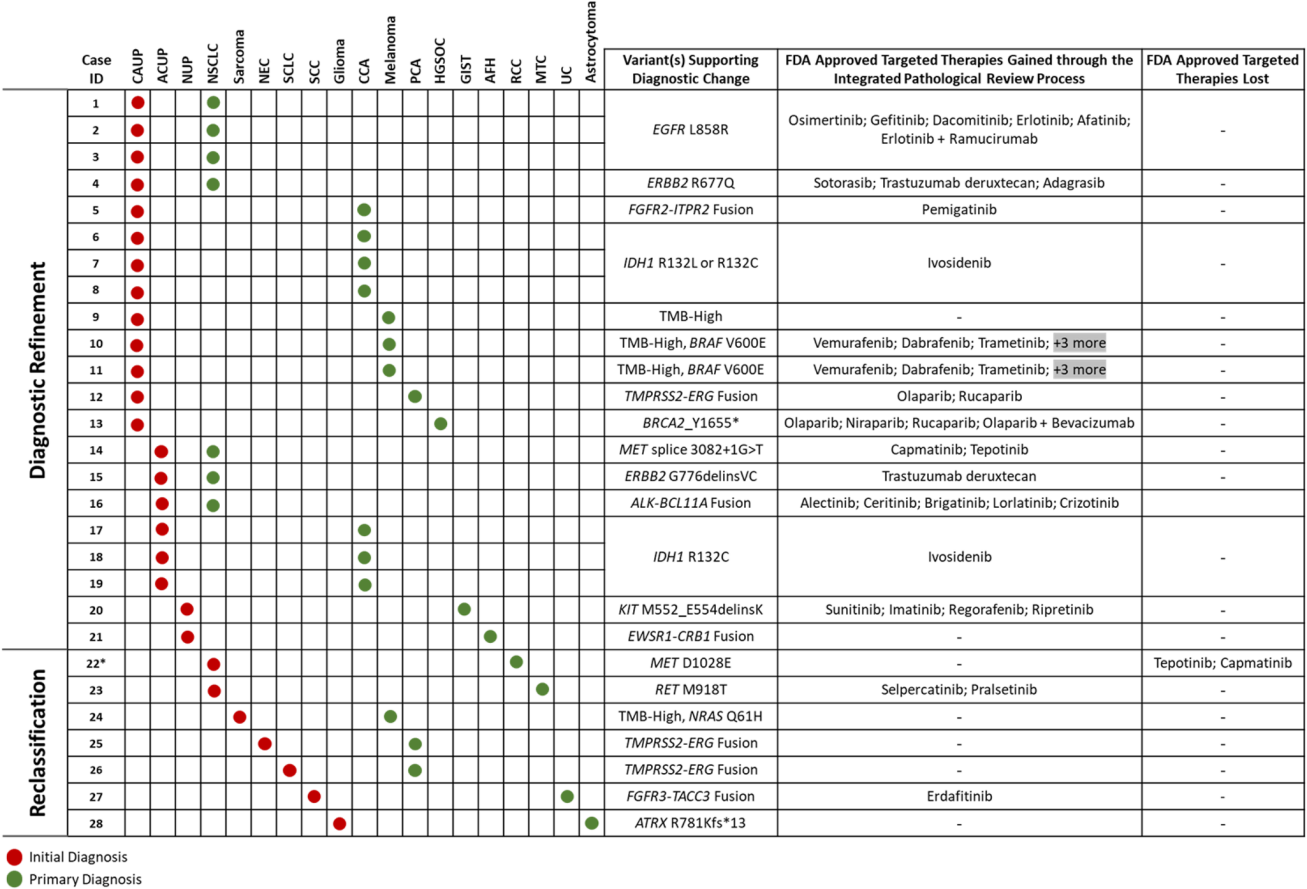
Detailed overview of patient cases that underwent diagnostic recharacterization based on contradictory primary pathological review and diagnosis and genomic findings ascertained from the PathGroup Endeavor NGS Test. For each case, initial diagnosis and either refined or reclassified final diagnosis are denoted with red and green dots, respectively. The variant(s) which initiated the secondary diagnostic review are provided as well as changes to targeted therapy options (FDA-approved targeted therapies gained or lost) due to the updated diagnosis and/or variant observation. For cases 10 and 11, additional FDA-approved targeted therapies gained include atezolizumab + vemurafenib + cobimetinib, vemurafenib + cobimetinib, and encorafenib + binimetinib. For cases 22, * denotes other critical findings, in addition to the *MET* D1028E variant conferring *MET* exon 14 skipping, that contributed to tumor reclassification. This included a likely germline *VHL* S111G alteration, which has been described in patients with Von Hippel Landau syndrome. Given the clinical history of renal cell carcinoma, young age, clear cell tumor morphology, and PAX8 IHC expression, this case was best classified as renal cell carcinoma (RCC) following the integrated review of clinical, immunohistochemical, molecular, and morphologic features. CAUP Carcinoma of unknown primary, ACUP Adenocarcinoma of unknown primary, NUP malignant neoplasm of unknown primary, NSCLC Non-small cell lung cancer, NEC Neuroendocrine carcinoma, SCLC small cell lung cancer, SCC Squamous cell carcinoma, CCA Cholangiocarcinoma, PCA Prostate carcinoma, HGSOC High-grade serous ovarian cancer, GIST Gastrointestinal stromal tumor, AFH Angiomatoid Fibrous Histiocytoma, RCC Renal cell carcinoma, MTC Medullary thyroid carcinoma, UC Urothelial carcinoma.

Comprehensive molecular profiling and completion of secondary pathological assessments resulted in disease reclassification or refinement for 28 cases presented in this study (See ‘Case Selection’ in Methods). Adult patients 18 years of age or older were included in this study with ages ranging from 19 to 83 years old (median 67.5) and 68% were male (Supplementary Table 1). Disease reclassification events are highlighted in seven cases in which initial diagnoses of non-small cell lung cancer (NSCLC; *n* = 2), sarcoma (*n* = 1), neuroendocrine carcinoma (NEC; *n* = 1), small cell lung cancer (SCLC; *n* = 1), squamous cell carcinoma (SCC; *n* = 1), and glioma (*n* = 1) were reclassified to final secondary diagnoses of renal cell carcinoma (RCC; *n* = 1), medullary thyroid carcinoma (MTC; *n* = 1), melanoma (*n* = 1), prostate carcinoma (PCA; *n* = 2), urothelial carcinoma (UC; *n* = 1), and diffuse astrocytoma (*n* = 1), respectively (Figs. 1 and 2). These cases represent scenarios where clinical presentation (e.g., anatomic site of metastasis) and/or preliminary pathology testing (e.g., cellular morphology) favored an initial diagnosis that was changed to a different diagnosis prompted by CGP results. Presented disease refinement events are comprised of 21 unique cases in which initial diagnoses of carcinoma of unknown primary (CaUP; *n* = 13), adenocarcinoma of unknown primary (ACUP; *n* = 6), or malignant neoplasm of unknown primary (NUP; *n* = 2) were refined to NSCLC (*n* = 7), cholangiocarcinoma (CCA; *n* = 7), melanoma (*n* = 3), PCA (*n* = 1), high-grade serous ovarian carcinoma (HGSOC; *n* = 1), gastrointestinal stromal tumor (GIST; n = 1), and angiomatoid fibrous histiocytoma (AFH; *n* = 1) (Figs. 1 and 2). These latter cases are demonstrative examples of clinical scenarios where an initial diagnosis could not be made prior to CGP, and in which CGP results were critical in refining a distinct diagnosis.

### Biomarkers driving diagnostic change impacting targeted therapy options

A wide variety of clinically informative genomic biomarkers were responsible for reclassification and refinement events, including SNVs, indels, gene fusions, and high tumor mutational burden (TMB-High). While all biomarkers have different levels of evidence and exclusivity of indication, these results were utilized in their respective clinical context to prompt secondary pathological assessment. Diagnostically informative biomarkers and their indicated diagnoses presented in the reclassification cohort were *RET* M918T (MTC), TMB-High (melanoma/NSCLC)^40^, *NRAS* Q61H (melanoma), *TMPRSS2-ERG* fusion (PCA), *FGFR3-TACC3* fusion (UC), and *ATRX* R781Kfs*13 (diffuse astrocytoma). For the disease refinement cohort, diagnostic biomarkers included *EGFR* L858R (NSCLC), *ERBB2* R677Q (NSCLC), TMB-High (melanoma/NSCLC), *FGFR2-ITPR2* fusion (CCA), *IDH1* R132L and R132C (CCA), *BRAF* V600E (melanoma), *CDKN2A* W110* (melanoma), *TMPRSS2-ERG* fusion (PCA), *BRCA2* Y1655* (HGSOC), *MET* exon 14 skipping (NSCLC), *ERBB2* G776delinsVC (NSCLC), *BCL11A-ALK* fusion (NSCLC), *KIT* M552_E554delinsK (GIST), and *EWSR1-CRB1* (AFH) (Fig. 2).

In addition to diagnostic evidence, the majority of the aforementioned biomarkers also served as therapeutic targets with FDA-approved targeted therapies indicated in the context of the newly defined disease indication. Of both reclassified and refined cohorts, 21 of the 28 patients gained at least one on-label targeted therapy that would not have been indicated under the primary diagnosis (Fig. 2). Gained therapies included tyrosine kinase inhibitors (TKIs) in the context of *EGFR-*mutated NSCLC, trastuzumab deruxtecan for *ERBB2*-mutated NSCLC, ivosidenib for *IDH1*-mutated CCA, MEK and RAF inhibitor combination therapies for *BRAF* V600E melanomas, and PARP inhibitors for *BRCA2*-mutated HGSOC. In only one case, where, upon secondary comprehensive review an initial diagnosis of NSCLC was updated to RCC, was an FDA-approved therapy lost, in the setting of a *MET* exon 14 skipping alteration (Fig. 2, case 22).

### CGP-driven patient outcome modeling

**Fig. 3:**
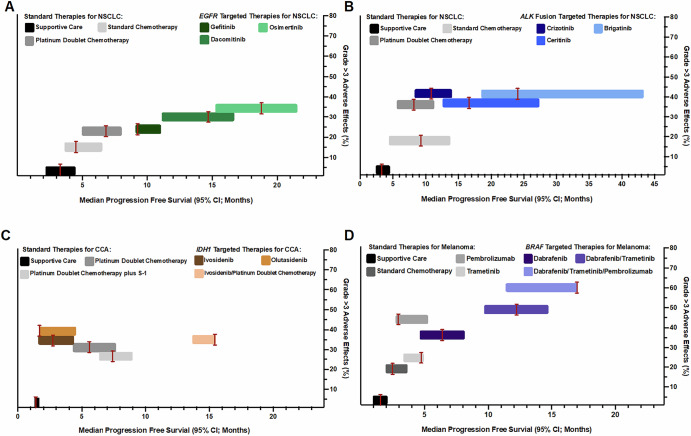
Therapy modeling outcomes evaluating median progression-free survival (PFS) and percent adverse events (AEs) for biomarker-targeted and standard, non-targeted therapies for NSCLC (A, B), CCA (C), and melanoma (D). PFS and AEs of grade >3 were plotted on x- and y-axes, respectively, to compare therapeutic efficacy and outcomes between supportive care, standard of care, and targeted therapies to explore the benefits of biomarker-based treatment decision making based on the updated final diagnoses of several cases in the study. An extensive review of completed clinical trials with published results was performed to identify relevant targeted therapies for *EGFR-*mutated NSCLC*, ALK-*rearranged NSCLC, *IDH1-*mutated CCA, and *BRAF* V600 mutation-positive melanoma, representing the most common observations in this cohort. For comparison, less stringent reviews of completed clinical trials focusing solely on the disease states, regardless of biomarker status, were performed to obtain patient outcome data representative of non-biomarker targeted therapies for NSCLC, CCA, and melanoma. In situations where either analysis failed to identify completed clinical trials with published results, a literature review was performed to identify appropriate data and references. References for all data utilized in therapy modeling analyses are listed in Supplementary Table 2. NSCLC, Non-small cell lung cancer; CCA, Cholangiocarcinoma.

While this case series highlights the important clinical utility of CGP in combination with pathological assessment to confirm diagnoses, projective modeling also illustrates the critical role CGP plays in predicting patient outcomes. Of the cases that were refined from CaUP to NSCLC, CGP results for three included the identification of the actionable biomarker, *EGFR* L858R. Detection of the *EGFR* biomarker not only prompted more accurate diagnoses, but also satisfied the FDA requirements for these patients to receive epidermal growth factor receptor tyrosine kinase inhibitor (EGFR-TKI) therapies. Compared to non-targeted therapies for *EGFR*-mutated NSCLC, such as standard or platinum-based chemotherapy, patients receiving either first (gefitinib) or third generation (osimertinib) EGFR-TKIs as first-line treatments showed significantly longer median PFS of 9.2 months (95% CI 9.1-11) and 18.9 months (95% CI 15.2-21.4), respectively, and superior OS (Fig. 3a**;** Supplementary Table 2). EGFR-TKIs therapies, regardless of generation, exhibited acceptable toxicities with patients reporting similar rates of serious (grade >3) adverse events (AEs) compared to standard and platinum-based chemotherapy, and no critical safety concerns were noted in either the AURA3 or FLAURA clinical trials^41^. One important outcome from the POSITION20 clinical trial was the determination that patients specifically harboring *EGFR* exon 20 insertions, which can be refractory to EGFR-TKIS, did achieve clinical results and increased median PFS of 9.7 months with a higher osimertinib dosing schema^42^. While CGP plays a fundamental role in therapy selection for patients, the utilizing of CGP to potentially refine patient treatment dosages is an interesting paradigm to consider. Substantial evidence has established EGFR-TKIs as the superior first-line therapy for patients with *EGFR*-mutated NSCLC, and clearly illustrates the importance of CGP in the guidance of treatment regimens and subsequent impact on patient care^43^.

Another critical biomarker that inferred NSCLC diagnosis among the study cases was the identification of anaplastic lymphoma kinase (*ALK*) fusions. Similar to several *EGFR* genomic variants, *ALK* fusions are actionable biomarkers that are targetable by ALK-TKI therapies, the preferred first-line treatment for *ALK*-positive NSCLC^44^. Patients receiving either first (crizotinib), second (alectinib, ceritinib, or brigatinib) or third generation (lorlatinib) ALK-TKIs showed significant gains in PFS compared to standard and platinum-based doublet chemotherapy with brigatinib exhibiting the highest median PFS of all therapies, 24.0 months (95% CI 18.5-43.2) (Fig. 3b, Supplementary Table 2). ALK-TKIs are highly tolerated, with comparable toxicities to standard and platinum chemotherapy, and result in longer intervals between therapy and the presentation of serious AEs or disease progression that require medical intervention^45^. While CGP enables *ALK* fusion detection and satisfies FDA requirements, it also offers additional screening for the presence or absence of secondary *ALK* mutations that may diminish the efficacy of ALK-TKIs and warrant a tailored patient treatment strategy that closely monitors for resistance and disease progression^46^.

Projective modeling also illustrated the critical role CGP plays in predicting patient outcomes in two CaUP cases that were refined to melanoma following the detection of the actionable biomarker *BRAF* V600E. Patients with *BRAF* V600 mutation-positive melanoma are eligible for treatment with FDA-approved BRAF inhibitors, though the current standard of care is immunotherapy^47^. Results from the DREAMSeq clinical trial determined that the best improvements in PFS and OS were in *BRAF* V600 mutation-positive melanoma patients receiving a combination of BRAF and MEK inhibitors (dabrafenib/trametinib) (Fig. 3c, Supplementary Table 2). While a safety and efficacy study examining varying combinations of BRAF, MEK, and PD-1 inhibitors (dabrafenib/trametinib/pembrolizumab) noted significant improvements in median PFS (17 months, 95% CI 11.3-NA) and OS (46.3 months, CI 23.9-NA) compared to single agent therapies, the study was terminated early due to the high number of participants who discontinued treatment as a result of experiencing serious AEs. As such, this remains a topic of interest in terms of sequencing therapeutic approaches in this space of targeted- and immune-based therapies.

In contrast to the previous cases studies, there are circumstances where CGP and identification of actionable biomarkers do not greatly improve upon standard therapies and patient outcomes. Of the seven cases that were refined from CaUP or ACUP to CCA, the CGP results for six included identification of the actionable biomarker *IDH1* R132L/C. Several IDH1 inhibitor therapies currently spanning two generations have FDA approval for treatment of *IDH1*-mutated CCA but have yet to demonstrate significant clinical outcome improvements compared to surgical resection and platinum-based doublet chemotherapy (Fig. 3d, Supplementary Table 2)^48^. One clinical trial investigating combination platinum-based doublet and IDH1 inhibitor therapies (cisplatin and gemcitabine plus ivosidenib) showed promising improvements of both median PFS (15.4 months, 95% CI 13.8-24) and OS (22.9, 95% CI 18.2-27) in patients with unresectable *IDH1*-mutated CCA. The study, however, was terminated prematurely due to low patient accrual (Supplementary Table 2)^48^. While CGP provided information critical for disease refinement and final diagnosis, the preferred first-line therapy following resection (if applicable) remains platinum-based doublet chemotherapy with no significant improvements to patient care identified during therapy modeling^49^. If CGP, however, can continue to assist with identifying these patients, this will likely facilitate patient accrual to meet baseline numbers necessary for future clinical studies.

## Discussion

CGP, based on its primary intended use, enhances patient care through biomarker detection, tumor characterization, diagnosis verification, and guidance towards therapeutic and clinical trial eligibility, and has been widely adopted by clinicians due to expanding guideline recommendations and reimbursement. This case series clearly demonstrates the clinical capability and application of CGP to more accurately define tumor diagnosis, as a secondary beneficial feature of this standard of care testing, in order to help resolve ambiguous or challenging cases and formulate personalized tumor treatment strategies, ultimately optimizing cancer patient care. In isolation, genomic biomarkers are not sufficient to render a diagnosis due to the potential overlap of occurrence, but in the context of favored differentials, they can significantly inform final decision making. In this cohort, all cases were re-evaluated based on conflicting pathological and molecular findings and assigned more accurate disease diagnoses, resulting in 75% (21/28) of cases gaining FDA-approved targeted therapies based on CGP results. While this study presents a refined cohort of cases for re-examination, the prevalence of actionable biomarkers among solid tumor types is substantial and warrants correlating to rates of CGP to better enable precision medicine strategies. As an example, *EGFR* mutations and *ALK* fusions are estimated to be present, on average, in 15% and 5% of NSCLC patients in the U.S., respectively, yet the clinical evidence illustrates that the rates of CGP testing is much lower than what is recommended based on clinical guidelines for patients with NSCLC^50–52^. Despite guideline advocacy for CGP, there continues to be high community dependence on single-gene testing (SGT) and small targeted panels^53^. While clinicians may prefer the quicker turnaround times, succinct interpretations, and improved coverage by commercial insurers of SGTs and small targeted panels, they have lower sensitivities and higher detection thresholds compared to CGP, often resulting in false negatives^54,55^. SGTs also lack the capabilities of providing detailed insight into tumor biology that may heavily influence patient disease diagnosis, treatment, and prognosis. Finally, in cases where tissue is limited, such as in NSCLC, SGT is an inefficient use of tumor material and therefore may lead to missed opportunities for comprehensive biomarker detection^56^.

The majority (75%) of cases highlighted in this series were initially classified as CUPs and included histotypes of carcinoma, adenocarcinomas, and malignant neoplasms of unknown primaries (CaUP, ACUP, and NUP, respectively). The prognoses of these ambiguous cases remain poor due to the lack of selective or indication-specific therapeutic options, defaulting most frequently to standard of care chemotherapy. Therefore, patients with these primary findings greatly benefit from CGP to refine diagnoses to identify predictive biomarkers of tumor origin, thus guiding physicians toward targeted therapies. One-third (7/21) of the CUP cases in this cohort were refined to NSCLC after secondary comprehensive clinicopathologic review of all results. Variants supporting the diagnostic change included clinically actionable *EGFR*, *MET*, and *ERBB2* mutations as well as *ALK* fusion, all of which are known oncogenic drivers that can be effectively treated with biomarker-matched targeted therapies. NSCLC is one of the most actionable solid tumor types with up to 50% of patients harboring actionable mutations^57^, which, when targeted, can improve patient OS^53^. The continued emergence of targeted therapies such as amivantamab and sunvozertinib for EGFR exon 20 insertions or lorlatinib in ALK-positive NSCLC represents a growing arsenal of effective therapeutic options^58–60^. Furthermore, with this new diagnosis, NSCLC patients can benefit from additional CGP to monitor disease for newly emerging biomarkers of clonality and resistance which may help guide subsequent lines of therapy. At the same rate, another third (7/21) of the CUP cases in this cohort were refined to CCA after secondary review, all of which, again, had clinically actionable biomarkers, including *IDH1* mutations or *FGFR2* fusion. These variant-based diagnostic changes led to patient eligibility for targeted therapies rather than standard of care chemotherapy alone or in combination with immune checkpoint inhibitors (ICIs), which have shown limited efficacy due to the aggressive nature of the cancer^61–63^. Of note, up to 50% of CCA cases have genomic alterations that may qualify patients for approved targeted therapies or agents that are currently under clinical investigation^64,65^. Despite this, there are limited indication-specific targeted treatment strategies for CCA that improve clinical outcomes beyond standard of care chemotherapy, although combination therapies hold some promise^66,67^.

Other CUP cases were refined to three cases of melanoma, and single cases of PCA, HGSOC, GIST, and AFH after an integrated clinicopathologic review of all findings. In the newly diagnosed melanoma cases, all exhibited TMB-High, the ultraviolet signature of this tumor type, which predicts responsiveness to anti-PD-1 and PD-L1 inhibitors otherwise known as ICIs^68,69^. Two of these cases also exhibited a *BRAF* V600E mutation, which provided the opportunity for these patients to qualify for a number of additional effective targeted therapies and treatment strategies^70–72^. In the cases updated to PCA, HGSOC, GIST, and AFH, only the AFH case did not gain an FDA-approved targeted therapy based on the diagnostic recharacterization. This low-malignancy potential tumor type does, however, often respond well to surgical resection and in more aggressive scenarios benefits from chemo- or radiotherapy^73^.

Of the seven reclassified cases, i.e., cases resulting in a change from one distinct indication to another, two were NSCLCs updated to RCC and MTC. In the case of the newly diagnosed RCC possessing a common *MET* D1028 mutation that confers *MET* exon 14 skipping^74^, FDA-approved targeted therapies, including tepotinib and capmatinib, which have demonstrated promising clinical activity in NSCLC, were lost due to the diagnostic update. These highly selective TKIs have received FDA approval within the last 2 years for NSCLC, and ongoing trials are assessing their efficacy in RCC^75^. The newly diagnosed *RET*-mutated MTC cases gained two FDA-approved therapies, which have been shown to have major efficacy as measured by improved overall response rate (ORR) and duration of response in the LIBRETTO-121 and ARROW trials^76,77^. Similarly, a sarcoma case was reclassified to melanoma, two histologies that are morphologically and immunohistochemically similar and oftentimes difficult to differentiate^78^. The genomic finding of TMB-High in these cases triggered the secondary pathology review as this finding is less common in sarcoma and is a defining genomic signature of melanoma. Although no targeted therapies were gained from this diagnostic update, ICIs have been shown to be therapeutically effective in patients with TMB-High melanoma. Two cases (NEC and SCLC) were reclassified as PCAs after a *TMPRSS2-ERG* fusion was identified via CGP. This fusion event is a defining diagnostic feature of PCA with up to 30% of patients exhibiting this biomarker^79^, and although it is not currently targetable by a precision therapy, it does help guide physicians towards a more specific treatment strategy tailored to prostate cancers. An SCC case originating from a prostate specimen was reclassified as a UC that had an actionable *FGFR3-TACC3* fusion that can be targeted by erdafitinib in the updated indication. This therapy has been shown to be more effective than standard of care chemotherapy with improvements in ORR, PFS, and OS^80^. Lastly, a glioma, which is an umbrella term used to describe different types of glial tumors^81^, was reclassified to astrocytoma, a more refined diagnosis, based on the presence of an *ATRX* frameshift mutation. Although no targeted therapies were gained with this reclassification, this differentiation can help guide standard of care modalities and predict outcomes.

In every case of diagnostic recharacterization, clinical decision making was made through a systematic and integrated review of all findings from histology to genomic results. With the large amount of available datapoints for each cancer patient, this comprehensive review process not only requires substantial interpretation by pathologists and oncologists but, in some scenarios, also the expertise of laboratorians, bioinformaticians, and molecular geneticists^82^. This highlights the importance of having a formalized institutional mechanism, i.e., molecular tumor board (MTBs), to aggregate this information in order to explore and align on therapeutic options to achieve optimized outcomes, and is particularly critical for deciphering challenging cases^83,84^. Through multidisciplinary MTBs, physicians identify key clinicopathologic features within a tumor that may predict therapeutic response, ultimately using an evidence-based approach to direct patients towards personalized strategies. Although oncologists most commonly attend and participate in MTBs, there has been an evolving and expanding role for diagnosticians and pathologists in these meetings to help guide the best course of action^85^ to optimize a cancer patients’ journey. This inclusivity has become even more essential with the clinical update of complex molecular testing, including NGS, which requires significant interpretation from multiple stakeholders. In the cases highlighted in this study, NGS results generated from a highly experienced reference laboratory were flagged as conflicting with the primary diagnosis, triggering a secondary review process to resolve the discrepancies. Although rare in occurrence, the integrated review helped refine over 20 CUP cases to new indication-specific diagnoses for which, in the majority of cases, FDA-approved targeted therapies were available. Furthermore, indication switching, that is, cases where a primary indication-specific diagnosis was updated to a distinctly different indication, occurred in seven cases, and although not all gained FDA-approved therapies, more optimized standard of care strategies could be pursued based on the new diagnoses.

While this case series highlights the role and value of CGP in diagnostic recharacterization, it has some limitations. Addressing one limitation and opportunity for future analysis, we identified 28 notable cases out of 10,000 total cases, giving the perception that diagnostic recharacterization triggered by contradictory clinicopathological and molecular findings is a rare occurrence. A more thorough and comprehensive retrospective review of all cases would inevitably identify additional cases that could have benefited from the integrated review process described in this study. Additionally, we have limited information about physician-enacted changes to clinical management following recharacterization. An interesting opportunity for future direction of this study is long-term clinical follow-up on the recharacterized cases to determine if therapeutic interventions based on the updated diagnoses were pursued and if improvements in patient outcomes were observed. In conclusion, CGP not only optimizes tissue stewardship while maximizing diagnostic and prognostic information to guide physicians towards targeted therapies, but also may help with confirming diagnosis to better identify therapeutic and trial options.

## Methods

### Patient case selection

Twenty-eight notable cases from adult patients (≥18 years) where tumor genomic profiling results were inconsistent with the initial pathological diagnosis and clinical presentation were selected from a robust cohort of more than 10,000 clinical cases that had previously received CGP from PathGroup in Nashville, TN. These cases were selected as representative examples of clinically impactful disease recharacterization events prompted by CGP results. Internal records for each patient were thoroughly reviewed for corresponding demographic information and all available immunohistological, molecular, and genetic test results. Case demographics that were present at the time patients underwent diagnostic recharacterization included patient age, sex, specimen site, and primary diagnosis. Additional demographics such as ethnicity, disease stage, and IHC, FISH, or other molecular results were conditionally available (Supplementary Table 1).

### Comprehensive genomic profiling

For genomic profiling, the PathGroup Endeavor NGS test, a pan-solid tumor, DNA-based CGP assay incorporating the FDA-cleared PGDx elio tissue complete test (Labcorp Oncology, Baltimore, MD, USA) was utilized to assess single nucleotide variants (SNVs) and insertion/deletions (indels) in 505 genes, gene amplifications in 28 genes, translocations in 23 genes, microsatellite status (microsatellite stable [MSS] or instable [MSI-H]), and tumor mutation burden (TMB)^7^. Sample processing from formalin-fixed paraffin-embedded (FFPE) tissue, including library preparation, hybrid capture, sequencing, and analysis was performed by PathGroup. Captured DNA libraries were sequenced on the NextSeq™ 550Dx platform (RRID:SCR_016384) using a high-capacity flow cell (Illumina, San Diego, CA, USA). Somatic variant identification was performed using the PGDx elio tissue complete bioinformatics software, which incorporates a machine learning-based variant calling algorithm to differentiate between true somatic mutations and artifacts or false positive signal^19^. Assignment of clinical significance to variants and clinical trial matching was performed using the Qiagen Clinical Insights service (Qiagen, Redwood City, CA, USA). PD-L1 IHC, using FDA-approved antibodies (RRID:AB_2889976) and an add-on to the Endeavor test, was also performed in the appropriate scenario specific to the site of origin.

### Histological review and other testing modalities

Tumor histology was available for review via a hematoxylin and eosin (H&E) slide or whole slide image. Additional clinical testing modalities for the presented cohort were performed by external laboratories or PathGroup and comprised of various IHC stains, FISH probe assays, DNA methylation sequencing, and RNA-based fusion detection via the Archer® Solid Tumor FUSIONPlex® assay (Integrated DNA Technologies, Coralville, IA, USA). Results from all testing modalities were included in the secondary integrated pathological review process when CGP results were misaligned with the primary diagnosis.

### Patient case review process for reconciling primary diagnoses and contradictory NGS-based findings

Prior to finalizing the CGP report, results were reviewed by both a board-certified laboratory director and a board-certified molecular pathologist. Presence (or absence) of a variant or variants with diagnostic implications (s), which either contradicted or further refined the diagnosis indicated at test initiation, triggered a secondary pathological assessment process. This integrated process involved a pathologist reviewing histology of the H&E slides, clinical presentation of the tumor, all concurrent testing results, and, when necessary, a consultation with members of the clinical team. Variants with disease-defining significance referenced in clinical guidelines or variants with strong disease-specific association were weighed to inform a differential diagnosis. If relevant, the specificity of immunostains utilized to infer the initial diagnosis was evaluated to confirm if the CGP-inferred diagnosis was definitively excluded. If a new consensus diagnosis was reached, the clinical significance of the detected variants was reinterpreted in the context of the new diagnosis to update the indicated therapeutic options and available clinical trials.

### Treatment selection and implementation practices with treating physicians

Assignment of tiers and levels of evidence for treatment options associated with the identified variants was conducted in alignment with AMP/ASCO/CAP interpretation guidelines^86^ and was assessed against therapies approved at the time testing was performed. Communication of diagnosis reclassification with treating physicians was facilitated through (1) the written CGP consultation section present on the front page of the issued CGP report, (2) direct contact with the treating physician, or (3) both.

### Therapeutic and clinical outcomes modeling

Therapy modeling was performed to illustrate theoretical gains in patient outcomes resulting from CGP biomarker detection and added eligibility for biomarker-targeted therapies. An extensive review of completed clinical trials with published results was performed to identify relevant FDA-approved targeted therapies for *EGFR-*mutated NSCLC*, ALK* fusion NSCLC, *IDH1-*mutated CCA, and *BRAF* V600 mutation-positive melanoma. For comparison, less stringent reviews of completed clinical trials focusing solely on the disease states, regardless of biomarker status, were performed to obtain patient outcome data representative of non-biomarker targeted therapies for NSCLC, CCA, and melanoma. In situations where either analysis failed to identify completed clinical trials with published results, a literature review was performed to identify appropriate data and references. While all clinical trials and guideline-recommended therapies could not be included in modeling, the resulting subset of clinical trials included demonstrates the diverse array of impacts therapy selection can have to patient care.

## Supplementary information

Supplementary Table 1,2

## Data Availability

The datasets generated and analyzed during the current study are not publicly available due to the proprietary ownership of the data and the privacy of the patient cohort but are available from the corresponding authors on reasonable request.

## Code availability

The datasets generated and analyzed during the current study are not publicly available due to the proprietary ownership of the data and the privacy of the patient cohort, but are available from the corresponding authors on reasonable request.
