# Supplementary material for: Tumor diagnosis recharacterization enabled by comprehensive genomic profiling to guide precision medicine strategy
Source: NPJ Precis Oncol. 2025 May 21;9:149. doi: 10.1038/s41698-025-00942-5 (PMC12095656; doi:10.1038/s41698-025-00942-5)
Supplement: Supplementary file 1 — Supplementary Table 1,2 [file 41698_2025_942_MOESM1_ESM.docx]

**Supplementary Table 1.** Overview of demographics and other test results (as available) for patient cases that underwent diagnostic recharacterization.

| **Case ID** | **Age (years)** | **Patient Sex** | **Ethnicity** | **Specimen Site** | **Primary Diagnosis** | **Stage** | **IHC Results** | **FISH Results** | **Other Molecular Results** |
| --- | --- | --- | --- | --- | --- | --- | --- | --- | --- |
| **1** | 77 | M | Caucasian | Lymph node | CaUP | IV | Pan-TRK: Negative | - | - |
| **2** | 50 | M | ND | Lung | CaUP | I | Pan-TRK: Negative | - | - |
|  |  |  |  |  |  |  | PD-L1: 80-100% cells 2-3+ |  |  |
| **3** | 82 | M | Caucasian | Peritoneal fluid | CaUP | Unknown | Pan-TRK: Negative | - | - |
|  |  |  |  |  |  |  | PD-L1: Low Expression |  |  |
| **4** | 61 | F | Caucasian | Thoracic spine | CaUP | IV | Pan-TRK: Negative | - | KRAS G12C |
|  |  |  |  |  |  |  | PD-L1: 1-2% cells 1-2+ |  |  |
| **5** | 62 | M | Caucasian | Liver | CaUP | II | Pan-TRK: Negative | - | FUSIONPlex: Negative |
|  |  |  |  |  |  |  | PD-L1: Negative |  |  |
| **6** | 75 | M | Caucasian | Liver | CaUP | Unknown | Pan-TRK: Negative | - | - |
| **7** | 47 | F | ND | Liver | CaUP | IV | Pan-TRK: Negative | - | - |
| **8** | 77 | F | Caucasian | Liver | CaUP | IV | - | - | - |
| **9** | 72 | M | Caucasian | Shoulder | CaUP | Unknown | Pan-TRK: Negative | - | FUSIONPlex: Negative |
|  |  |  |  |  |  |  | PD-L1: 40-50% cells 2-3+ |  |  |
|  |  |  |  |  |  |  | MSI: Normal |  |  |
| **10** | 51 | F | Hispanic | Lower leg | CaUP | Unknown | Pan-TRK: Negative | - | - |
|  |  |  |  |  |  |  | PD-L1: 2% cells 2+ |  |  |
| **11** | 42 | M | Caucasian | Brain Tissue | CaUP | IV | Pan-TRK: Negative | - | - |
|  |  |  |  |  |  |  | PD-L1: 70-80% cells 2-3+ |  |  |
| **12** | 71 | M | Caucasian | Mediastinum | CaUP | III | NKX3.1: Patchy positive | - | BRCA2 intron 7 and exon 24 truncation (deleterious) |
|  |  |  |  |  |  |  | Pan-TRK: Negative |  |  |
|  |  |  |  |  |  |  | PD-L1: 1-10% cells 2-3+ |  |  |
| **13** | 64 | F | ND | Peritoneal fluid | CaUP | Unknown | FOLR1: 1-5% cells 0-1+ | - | - |
|  |  |  |  |  |  |  | PD-L1: <1% cells 0-1+ |  |  |
| **14** | 83 | M | Caucasian | Right face | ACUP | IV | PD-L1: 10-20% cells 3+ | - | - |
| **15** | 68 | F | ND | Chest wall | ACUP | IV | CK7: Positive | - | - |
|  |  |  |  |  |  |  | CK20: Negative |  |  |
|  |  |  |  |  |  |  | Napsin A: Positive |  |  |
|  |  |  |  |  |  |  | Pan-TRK: Negative |  |  |
|  |  |  |  |  |  |  | PD-L1: 50-75% cells 2+ |  |  |
|  |  |  |  |  |  |  | TTF-1: Positive |  |  |
| 16 | 81 | M | ND | Abdominal wall | ACUP | Unknown | AE1/AE3: Positive | - | - |
|  |  |  |  |  |  |  | Calretinin: Negative |  |  |
|  |  |  |  |  |  |  | CDX2: Negative |  |  |
|  |  |  |  |  |  |  | CK7: Cytoplasmic staining |  |  |
|  |  |  |  |  |  |  | CK20: Negative |  |  |
|  |  |  |  |  |  |  | GATA3: Negative |  |  |
|  |  |  |  |  |  |  | NKXS.1: Negative |  |  |
|  |  |  |  |  |  |  | P504S: Negative |  |  |
|  |  |  |  |  |  |  | PSA: Negative |  |  |
|  |  |  |  |  |  |  | TTF-1: Positive |  |  |
|  |  |  |  |  |  |  | WT1: Negative |  |  |
| **17** | 75 | M | Caucasian | Liver | ACUP | II | - | - | - |
| **18** | 64 | F | Caucasian | Sigmoid colon | ACUP | IV | MSI: Normal | HER2: Negative | - |
|  |  |  |  |  |  |  | Pan-TRK: Negative |  |  |
|  |  |  |  |  |  |  | PD-L1: Negative |  |  |
| **19** | 67 | F | Other | Liver | ACUP | IV | Pan-TRK: Negative | - | - |
| **20** | 70 | F | ND | Liver | NUP | IV | - | - | - |
| **21** | 19 | M | ND | Brain Tissue | NUP | Unknown | - | EGFR Amp: Negative | - |
|  |  |  |  |  |  |  |  | PTEN Loss: Negative |  |
| **22** | 56 | M | Caucasian | Lung | NSCLC | Unknown | Pan-TRK: Negative | - | VHL S111G |
| **23** | 70 | M | ND | Bone | NSCLC | Unknown | PD-L1: 60-80% cells 2-3+ |  | ROS1: Negative |
| **24** | 74 | M | Caucasian | Lymph node | Sarcoma | IV | Pan-TRK: Negative | - | FUSIONPlex:Negative |
|  |  |  |  |  |  |  | PD-L1: 60-80% cells 2-3+ |  |  |
| **25** | 69 | M | Caucasian | Breast | Neuroendocrine carcinoma | Unknown | Pan-TRK: Weakly positive | - | - |
|  |  |  |  |  |  |  | PD-L1: <1% cells 0-1+ |  |  |
| **26** | 54 | M | Caucasian | Lymph node | Small cell carcinoma | IV | CK 8/18: Positive | HER2: Negative | - |
|  |  |  |  |  |  |  | Pan-TRK: Negative |  |  |
|  |  |  |  |  |  |  | PD-L1: Negative |  |  |
|  |  |  |  |  |  |  | TTF-1: Positive |  |  |
| **27** | 82 | M | Caucasian | Prostate | Squamous cell carcinoma | Unknown | Pan-TRK: Negative | - | - |
|  |  |  |  |  |  |  | PD-L1: 1-10% cells 1- 2+ |  |  |
| **28** | 57 | M | ND | Spinal cord | Glioma | III | - | EGFR Amp: Negative | - |
|  |  |  |  |  |  |  |  | PTEN Loss: Negative |  |
|  |  |  |  |  |  |  |  | Polysomy: Positive (chromosome 1,7,10,19) |  |
|  |  |  |  |  |  |  |  | Trisomy 9: Positive |  |

(-) or ND (not recorded) denote missing and/or unavailable information at the time of analysis. CaUP, Carcinoma of unknown primary; ACUP, Adenocarcinoma of unknown primary; NUP, malignant neoplasm of unknown primary; NSCLC, non-small cell lung cancer; Fluorescence *in situ* hybridization, FISH; Immunohistochemistry, IHC.

**Supplementary Table 2.** Summary of chemotherapy, platinum chemotherapy, immune check point inhibitor, and biomarker targeted therapy clinical trials used in therapy modeling analyses

| **Clinical Trial Cohort** | **Therapy** | **Trial Identifier** | **Year** | **Phase** | **# of Participants** | **Reference** |
| --- | --- | --- | --- | --- | --- | --- |
| Treated (1 prior chemotherapy regime) NSCLC, *EGFR* status unknown | None | NCT00606021 | 2011 | II | 27 | [74] |
|  | Pemetrexed or Docetaxel | NCT00556322 | 2015 | III | 221 | [75] |
| Untreated NSCLC, *EGFR* status unknown | Pemetrexed/Cisplatin | NCT00949650 | 2014 | III | 115 | [76] |
| Untreated NSCLC, *EGFR-m*utated | Gefitinib | NCT01774721 | 2022 | III | 227 | [77] |
|  | Dacomitinib | NCT01774721 | 2022 | III | 225 | [77] |
|  | Osimertinib | NCT02296125 | 2017 | III | 350 | [78] |
| Treated (1 prior chemotherapy regime) NSCLC, *ALK* fusion status  unknown | None | NCT00606021 | 2011 | II | 27 | [74] |
|  | Pemetrexed | NCT00191191 | 2008 | II | 226 | [79] |
| Untreated NSCLC, *ALK-*rearranged | Pemetrexed/Cisplatin | NCT01828099 | 2024 | III | 187 | [80] |
|  | Ceritinib | NCT01828099 | 2024 | III | 189 | [80] |
|  | Crizotinib | NCT01154140 | 2016 | III | 172 | [81] |
| Mixed untreated and treated (≤ 1 prior chemotherapy regime)  NSCLC, *ALK-*rearranged | Brigatinib | NCT02737501 | 2021 | III | 137 | [82] |
| Treated (1 prior chemotherapy or 5-FU regime) CCA, *IDH1-*mutated | None | NCT02989857 | 2021 | III | 61 | [83] |
|  | Ivosidenib | NCT02989857 | 2021 | III | 126 | [84] |
|  | Olutasidenib | NCT03684811 | 2022 | I/II | 32 | [85] |
|  | Ivosidenib/Cisplatin/Gemcitabine | NCT04088188* | 2023 | I | 6 | [48] |
| Untreated metastatic melanoma, *BRAF* status unknown | None | - | - | - | - | [86] |
| Untreated metastatic melanoma, mixed wild-type and *BRAF-*mutated | Dabrafenib | NCT00864253 | 2014 | III | 265 | [87] |
| Mixed untreated and treated (≤1 prior chemotherapy regime)  metastatic melanoma, *BRAF-*mutated | Trametinib | NCT01245062 | 2016 | III | 214 | [88] |
| Treated (1 prior chemotherapy regime) metastatic melanoma, mixed  wild-type and *BRAF-*mutated |  |  |  |  |  |  |
| Untreated metastatic melanoma, *BRAF-*mutated | Pembrolizumab | NCT01704287 | 2019 | II | 361 | [89] |
|  | Dabrafenib | NCT01584648 | 2019 | III | 212 | [90] |
|  | Dabrafenib/Trametinib | NCT01597908 | 2019 | III | 352 | [91] |
|  | Dabrafenib/Trametinib/Pembrolizumab | NCT02130466 | 2021 | I/II | 75 | [92] |

(-) denote agents that have not yet been evaluated in clinical trials. (*) denote clinical trials that were terminated prior to the proposed completion date. NSCLC, non-small cell lung cancer; CCA, Cholangiocarcinoma.
